# Double-Blind Randomized Phase 2 Trial Testing Personal Cancer Vaccines in Patients with Advanced Ovarian Cancer

**DOI:** 10.3390/vaccines13111099

**Published:** 2025-10-28

**Authors:** Lisa N. Abaid, Bradley R. Corr, Ramez N. Eskander, James R. Mason, Katrina L. Lopez, Krystal Godding, Rockelle M. Robles, Hans S. Keirstead, Gabriel I. Nistor, Robert O. Dillman

**Affiliations:** 1 Hoag Hospital Gynecologic Oncology, Newport Beach, CA 92663, USA; labaid@gmail.com; 2 University of Colorado Cancer Center, Aurora, CO 80045, USA; bradley.corr@ucdenver.edu; 3 University of California San Diego Moores Cancer Center, La Jolla, CA 92037, USA; reskander@ucsd.edu; 4 Scripps Clinic, La Jolla, CA 92121, USA; mason.james@scrippshealth.org; 5 AIVITA Biomedical, Inc., Irvine, CA 92612, USA; katrina@aivitabiomedical.com (K.L.L.); krystal@aivitabiomedical.com (K.G.); rockelle@aivitabiomedical.com (R.M.R.); hans@aivitabiomedical.com (H.S.K.); gabriel@aivitabiomedical.com (G.I.N.)

**Keywords:** ovarian cancer, dendritic cell vaccine, autologous tumor antigens

## Abstract

**Background/Objectives:** Dendritic cell vaccines are a promising cancer immunotherapy. AV-OVA-1 is a patient-specific vaccine consisting of autologous dendritic cells (DCs) loaded with autologous tumor antigens (ATA) from a lysate of irradiated self-renewing cells enriched for tumor-initiating cells (TICs). A multicenter, double-blind, randomized phase 2 trial was designed to determine manufacturing feasibility, safety, and efficacy. **Methods:** Patients had newly diagnosed stage 3 or 4 ovarian cancer. Short-term cell cultures were established from freshly resected tumor specimens. Patients were screened for randomization seven months after initial diagnosis, after completing neoadjuvant and/or adjuvant chemotherapy and surgery. Eligibility included a successful cell culture, cryopreservation of sufficient monocyte numbers for differentiation into DCs, and good performance status. Patients were stratified by whether they had persistent disease; then, they were randomized 2:1 to AV-OVA-1 or autologous monocytes (MC). Cryopreserved doses of AV-OVA-1 and MC were thawed and admixed with granulocyte–macrophage colony-stimulating factor just before subcutaneous injections at weeks 1, 2, 3, 8, 12, 16, 20, and 24. **Results**: Study accrual was terminated early during the SARS-CoV-2 pandemic. Manufacturing success rates for TICs, monocyte intermediate products, and AV-OVA-1 were 70/72 (97.2%) and 47/50 (94.0%), and 29/30 (96.7%), respectively. A total of 29 participants were treated with AV-OVA-1 and 15 with MC. Patients received an average of 7.4 injections. Adverse-event frequencies were similar in both arms, mild to moderate in severity, and self-limited. T-cell immune responses increased only after AV-OVA-1. There were no survival differences in this underpowered study. **Conclusions:** AV-OVA-1 was manufactured reliably and injections were well tolerated.

## 1. Introduction

Ovarian cancer is a major health problem. Globally, in 2020, ovarian cancer was the eighth most common malignancy and the eighth most common cause of death in females [1]. In the United States, in 2025, it was the 11th most common cancer, but the 6th most common cause of cancer death in women [2]. A total of 75% of ovarian cancers are advanced disease at diagnosis, 67% classified as stage 3 and 33% as stage 4 [3]. For several decades, the standard management of newly diagnosed advanced ovarian cancer has included surgical resection and platinum-taxane chemotherapy [4]. In the past decade, recent additions to the therapeutic armamentarium included the anti-vascular endothelial growth factor monoclonal antibody bevacizumab [5,6,7], and various poly (ADP-ribose) polymerase (PARP) inhibitors [8,9]. Neoadjuvant therapy is preferred for patients who cannot be readily rendered disease-free by surgery, even though randomized clinical trials show no difference in progression-free survival (PFS) or overall survival (OS) between standard adjuvant therapy following primary surgical debulking compared to neoadjuvant plus adjuvant therapy (interval debulking) [10,11]. Despite these measures, the relative 5-year survival rate was only 30% for patients diagnosed with advanced ovarian cancer during both 2011–2017 and 2014–2020 [1,12].

Adding adjuvant immunotherapy to standard ovarian cancer therapy may be beneficial [13], but adding immune checkpoint inhibitors has not improved survival [14,15]. Another adjuvant immunotherapy strategy is adding a vaccine. Because of biological heterogeneity and neoantigens resulting from non-synonymous mutations [16], autologous tumor antigens (ATAs) have become the antigens of choice for such vaccines, even though they are more complicated to manufacture compared to off-the-shelf vaccine products [17,18]. One promising personalized vaccine strategy uses autologous dendritic cells (DCs) loaded ex vivo with ATA [19,20]. DC-ATA products have consisted of DC derived from peripheral blood mononuclear cells and loaded with autologous whole tumor lysates [21,22,23,24,25], mRNA from autologous whole tumor [26,27,28], and antigens from autologous irradiated proliferating cancer cells [29,30,31,32,33]. In addition to using unique, patient-specific antigens, these ATA approaches include any tumor-associated antigens that are shared among patients.

The last approach is unique in that the antigen source is self-renewing cells grown in short-term cell culture conditions that favor tumor-initiating cells (TICs), including stem cells and early progenitor cells. TICs constitute only a small fraction of cells in a tumor, but are believed to be largely responsible for metastasis and resistance to anti-cancer therapies [20,34]. This unique DC-ATA treatment can be used for any cancer, but because a fresh tumor is needed to establish self-renewing cell cultures, it has been studied mostly in malignancies in which surgery is a standard treatment modality. Because this DC-ATA product is autologous and contains no additives or excipients, there is no theoretical basis for allergic reactions. It has been well tolerated in hepatocellular cancer [29], renal cell cancer [30], melanoma [31,32], and glioblastoma (GBM) [33]. Efficacy was suggested by delayed complete tumor regressions in 10% of patients [20,30,32], by a 50% increase in PFS compared to historical controls in GBM [33], and a more than doubling of median OS in melanoma patients compared to historical and randomized controls [31,32].

Ovarian cancer TICs are an attractive therapeutic target for vaccine-induced T cells [35,36,37,38,39]. Newly diagnosed advanced epithelial ovarian cancer is an ideal clinical setting in which to treat with this DC-ATA because extensive surgical debulking is a component of standard treatment, and the current 5-year survival rate is unsatisfactory. AV-OVA-1 consists of autologous DC loaded with antigens from self-renewing ovarian cancer cells. This article reports a multi-center randomized phase 2 clinical trial designed to determine the feasibility of manufacturing AV-OVA-1 and the safety and efficacy of injecting AV-OVA-1 into patients with advanced disease.

## 2. Methods

### 2.1. Trial Design and Oversight

**Figure 1:**
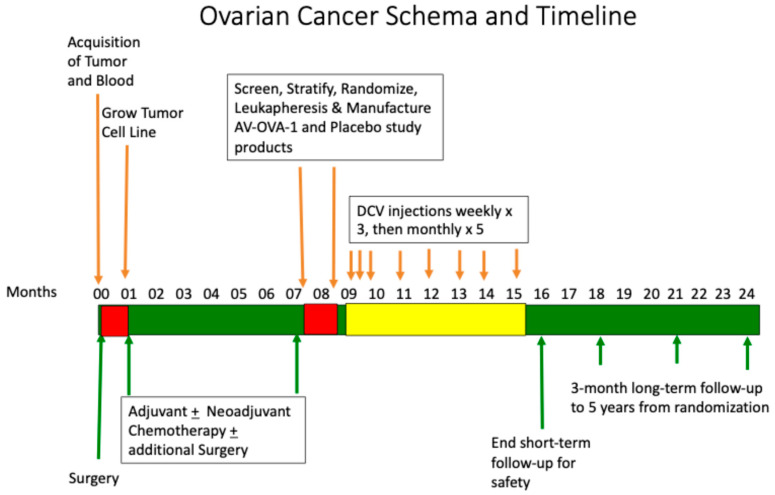
Schema and timeline for patient enrollment, tumor acquisition, tumor cell culture, completion of standard treatment, screening and intent-to-treat enrollment, randomization, manufacturing of study product, treatment, and follow-up.

The study design is shown in Figure 1. A detailed schematic of the manufacturing of such DC-ATA products was previously published [20]. This multi-center, 2:1 double-blind, randomized phase 2 clinical trial compared adjuvant AV-OVA-1 to placebo (autologous monocytes, MC) and was conducted in patients with advanced ovarian cancer enrolled from four academic centers and two community centers in the western United States (ClinicalTrials.gov NCT00331526, registered 1 September 2014). Treating physicians were gynecologic oncologists. The trial was conducted per the principles of the Declaration of Helsinki and Good Clinical Practice, and with local approval and oversight by review boards for all six participating institutions. An independent monitoring committee reviewed trial data on an annual basis. Before surgery, participants gave written informed consent for the collection of tumor tissue and blood. After completing primary therapy, about seven months after the initial diagnostic surgery, a second informed consent was obtained to permit screening and randomization for treatment assignment. Study treatment injections began about nine months following initial surgery.

### 2.2. Patients

#### 2.2.1. Eligibility for Tumor Collection

Key eligibility criteria for tumor collection were: (1) suspected new primary stage 3 or 4 epithelial ovarian cancer, (2) age > 18 years, (3) tentative willingness to later undergo a leukapheresis to obtain peripheral blood mononuclear cells (PBMC), and (4) written informed consent.

#### 2.2.2. Eligibility to Screen for Randomization

Key eligibility for randomization were: (1) confirmed primary stage 3 or 4 ovary cancer, (2) a successful tumor cell line and ATA lysate, (3) collection and cryopreservation of sufficient numbers of monocytes for MC placebo or differentiation into DC, (4) completion of surgery and adjuvant/neoadjuvant platinum-based chemotherapy, (5) Eastern Cooperative Oncology Group PFS of 0 or 1, (6) characterization of extent of disease at time of screening: no evidence of disease or evidence of disease (including elevated CA125), and (7) written informed consent. Key exclusion criteria were underlying disease processes considered life-threatening within five years, active infection or other ongoing medical condition deemed to be potentially life-threatening, or nursing or pregnancy.

#### 2.2.3. Stratification and Randomization

After completing all adjuvant and/or neoadjuvant chemotherapy, patients were screened for eligibility for randomization, and if eligible, stratified based on whether they had residual disease, then assigned a study product by the laboratory quality manager based on a random number-generated table with 2:1 randomization to AV-OVA-1 or MC with a six-block size.

### 2.3. Manufacturing of AV-OVA-1 and MC

#### 2.3.1. Autologous Tumor Antigens

The source of ATA was a lysate of irradiated autologous tumor cells from a short-term cell culture of each patient’s ovarian cancer. Fresh ovarian cancer tissues collected during surgery were placed into transport medium provided by the sponsor, then transported to AIVITA Biomedical, Inc. (Irvine, CA, USA) by overnight courier. Tumors were mechanically dissected and enzymatically digested to produce single-cell suspensions, which were incubated in proprietary media supplemented with fibroblast growth factor and epidermal growth factor to promote spheroid growth, which is associated with enrichment for TICs. These culture conditions eliminate stromal cells, connective and vascular tissues, as well as lymphocytes and other hematopoietic cells. Tumor cell cultures were expanded to at least 100 million cells, or for 28 days, whichever occurred first. MUC-1 and CA-125 expression on cultured cells was determined using immunocytochemistry stains. Tumor cells were treated with 100 Gy (Precision CellRad x-ray cell irradiator, Precision X-Ray, Branford, CT, USA), and then the “stressed” tumor cells were cultured for 18 to 24 h to enable the expression of damage-associated molecular proteins. Next, these cells were frozen and repeatedly thawed to yield a tumor cell lysate that was then cryopreserved in liquid nitrogen.

#### 2.3.2. Monocytes and Autologous Dendritic Cells

PBMC were collected by leukapheresis and shipped to the AIVITA manufacturing facility by overnight courier. Only 4/45 patients underwent leukapheresis before starting chemotherapy. The Elutra^®^ Cell Separation System (Terumo BCT, Lakewood, CO, USA) was used to enrich the PBMC product for monocytes (MC). Additional leukapheresis procedures were allowed if the MC number was less than 450 million. MCs were cryopreserved until randomization, when, depending on treatment assignment, the MCs were differentiated into DCs or retained for aliquoting into doses to serve as a placebo control. For the AV-OVA-1 arm, cryopreserved MCs were differentiated into DCs by incubating for six days in media supplemented with the cytokines interleukin-4 (CellGenix, Portsmouth, NH, USA) and GM-CSF (Leukine^®^, Partner Therapeutics, Lexington, MA, USA). Differentiation of MCs into DCs was confirmed by a phenotype that was CD14-, CD11c+, CD83+, CD86+, HLA-I+, and HLA-II+, with smaller percentages of cells that were CD141+ and CCR7+.

#### 2.3.3. AV-OVA-1 (AV-OVA-1) and MC

Treatment study products were manufactured following randomization. After six days of differentiating MC into DC, patient-specific AV-OVA-1 vaccines were produced by incubating autologous DC for 18 to 30 h with the patient’s cryopreserved ATA lysate. Release criteria for AV-OVA-1 included negative tests for endotoxin and *Mycoplasma* or microbial contamination. and. Each AV-OVA-1 or MC product was subdivided into 10 equivalent aliquots, and then the vials were stored at −190 °C to −150 °C in liquid nitrogen. Individual patient doses of viable AV-OVA-1 pre-cryopreservation ranged from 2.0 to 27 million (M) cells with a median of 7.7 M, a mean of 7.7 ± 5.6 (StDev) M and interquartile range (IQR) 3.8 to 12.0 M. Individual patient doses of viable MCs post-cryopreservation ranged from 8.0 to 24.6 million (M) cells with a median of 9.2 M, a mean of 12.0 ± 5.1 M, and IQR 8.9 to 12.2. M. Individual doses were shipped via liquid nitrogen dewar to the treatment location. At the site’s pharmacy, with blinding, the study product was thawed, suspended in 500 μg GM-CSF (Leukine^®^), followed by the s.c. injection within five hours of thawing.

### 2.4. Treatment

Patients were randomly assigned in a 2:1 ratio to receive AV-OVA-1 or MC. Blinded, coded trial regimen kits masked the assigned agent. Patients, managing physicians, caregivers, and pharmacists were all blinded to the treatment. Study agents were injected beginning eight to nine weeks after manufacturing. Injections were scheduled for weeks 1, 2, 3, 8, 12, 16, 20, and 24. Patients were allowed to receive other standard therapies concurrently with the study agent, but other investigational agents were not permitted during study treatment. After completing the vaccine, there were no treatment restrictions.

### 2.5. Endpoints

The primary endpoint was OS calculated from randomization. Secondary endpoints were PFS from randomization, OS and PFS from the date at which surgical tissue was originally obtained, and manufacturing success rates for tumor cells, monocytes, and a successful DC-ATA product.

Safety endpoints included serious adverse events (SAEs) and the frequency, duration, and severity of treatment-emergent adverse events (AEs) that were identified and classified per the National Cancer Institute Common Terminology Criteria for Adverse Events (NCI-CTCAE v 4.03). Investigators classified AE as “related,” possibly related,” or “unrelated” to the study product. Baseline history and physical examination were documented during screening for eligibility and randomization. Cursory clinical assessments were documented at each treatment visit. Serum chemistry tests, complete hematologic cell counts, and computerized axial tomography (CAT) and photon emission tomography (PET) scans were ordered per standard of care. The final assessment for safety took place 28 days after the final vaccine injection. Thereafter, follow-up assessments were obtained at 3-month intervals from randomization. At each treatment location, trial data were entered by research personnel into a research electronic data capture database (REDcap^®^, version 1.4, REDcap Cloud, Encinitas, CA, USA) [40].

A tertiary endpoint was the in vivo T-cell immune response as detected with an enzyme-linked immunosorbent spot (ELISpot) assay for intracellular interferon-gamma (IFN-γ). Heparinized blood samples were obtained just before each of the first four vaccine injections (day-0 baseline and days 7, 14, and 56). PBMC were isolated by Ficoll–Hypaque density centrifugation and cryopreserved. Subsequently, samples were thawed and analyzed for the 39 patients who had baseline samples, and for the 34 patients who received at least the first three weekly vaccinations and had blood samples available for analysis. PBMC were incubated for seven days with IL-2 in AIM5 medium (Thermo Fisher, Waltham, MA, USA), then transferred to 12-well plates containing IL-2 and AIM5, then incubated for two more days. On day 9 the cells were transferred to 96-well IFN-y ELISpot plates (Bio-Techne Corp, Minneapolis, MN, USA). After incubating 20 min at room temperature in 200 mL of AIM5, 5 mL of phytohemagglutinin was added to 3 wells as a positive control. Samples were incubated overnight in a humidified 37 °C CO_2_ incubator, then 200 mL of anti-IFN-γ antibody detection reagent was added to each well, then the plates were incubated for two hours at room temperature. After washing, 100 μL of diluted Streptavidin-AP Concentrate A was added to each well and incubated for 2 h at room temperature, then washed, and 100 μL of BCIP/NBT color development substrate (Thermo Fisher, Boston, MA, USA) was added to each well, then incubated for one hour in the dark. All liquid was then decanted, and the plates were dried for 90 min. Each well was then examined at 4× magnification under a light microscope. The number of darkly stained spots in each well was calculated using a hemocytometer. Results were reported as the number of ELISpots per 150,000 cells counted. At baseline, expression of 10 or more ELISpots per 150,000 was considered positive. An increase was considered significant if the average number increased by 25% or more and by at least seven ELISpots. All assays were performed by the same technician.

### 2.6. Statistical Methods

The study size of 99 patients (66 AV-OVA-1, 33 MC) was based on a desired 50% reduction in deaths in the AV-OVA-1 arm compared to MC, with an alpha of 0.20, power of 80%, a 2:1 randomization, projected 36-month median OS in the control arm, and 10% lost to follow-up. It was estimated that tumors from 110 patients would be needed to randomize 99. Analysis was planned after 43 deaths, which were estimated to occur after five years of follow-up. Efficacy analysis was based on the intent-to-treat (ITT) population (all patients who underwent randomization). Safety analyses were based on treatment received.

Clinical trial data was collected at each site and entered into an electronic data capture system [37]. Missing data was not imputed. Baseline characteristics were collected at randomization. Death by any cause was used to define the death endpoint. The date of disease progression was defined by death or designated by the principal investigator/managing physician based on clinical, CA-125, and radiographic changes. No specific radiologic criteria were prescribed for defining PD, and there was no central review of scans, but the basis for declaring PD was recorded. The numbers of AEs by tissue classification and severity grade were tabulated per NCI-CTCAE v 4.03 for all patients who received at least one study agent injection.

Continuous and categorical variables were characterized using descriptive statistics. Means were compared via T-tests. Proportions were compared by either the Chi-square test or Fisher’s Exact Test. Manufacturing efficiency was calculated as the percentage of successes per the number of patients for whom an attempt was made to manufacture an intermediate or final study product. PFS and OS curves were generated using the Kaplan–Meier method, and were compared using unadjusted Mantel–Cox log-rank tests. Graphs were generated using GraphPad Prism 10.4. No adjustments were made for multiple analyses.

## 3. Results

### 3.1. Study Conduct

**Figure 2:**
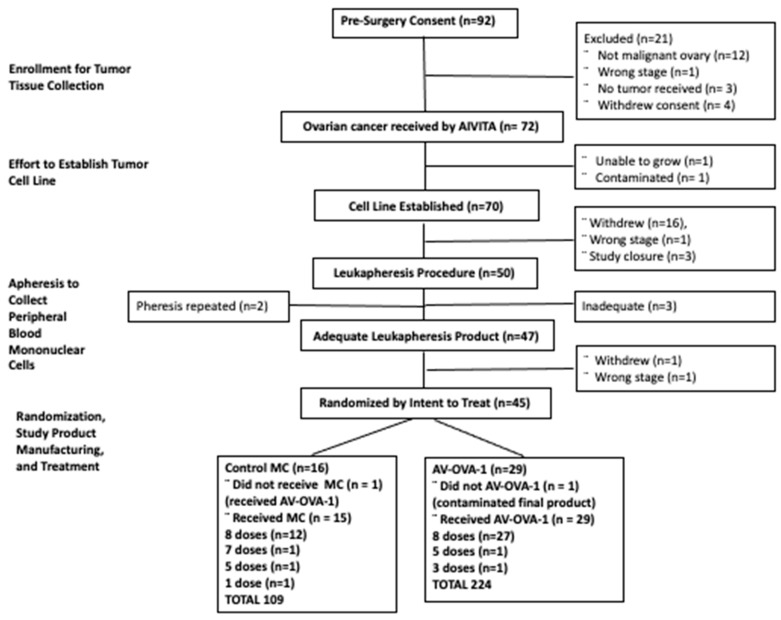
CONSORT flow chart.

The accompanying CONSORT flow chart (Figure 2) shows the trial’s conduct from screening for tumor collection through randomization and treatment. Patients were enrolled from five sites in California and one in Colorado, with tumors collected from 92 patients during the 41 months between December 2017 and April 2021. Randomization occurred between May 2018 and June 2021. A total of 45 patients were randomized, 29 to AV-OVA-1 and 16 to MC. As shown in the CONSORT Diagram, screening 92 patients resulted in collecting 72 ovarian epithelial cancers that yielded 70 successful cell lines, 47 sufficient leukapheresis products, and randomization of 45 patients for a 48.9% efficiency rate of randomization based on initial screening, and randomization of 45/47 (95%) of those for whom a cell line and sufficient monocytes were available. The major reasons for not proceeding with protocol-prescribed procedures were patient withdrawal of consent (22.8%), not having ovarian cancer at the time of surgery (13.0%), not stage 3 or 4 (3.3%), no tumor submitted (3.3%), insufficient monocytes collected (3.3%), premature study closure (3.3%), and no tumor cell line (2.2%).

There were no differences between arms in the time from surgery to randomization, nor from surgery or randomization to the first injection of a study agent. The mean and median times from surgery to randomization were, respectively, 6.1 and 5.9 months in the AV-OVA-1 arm (range 3.2 to 15.1) and 6.4 and 6.3 months in the MC arm (range 3.1 to 8.4). The mean and median months from surgery to the first treatment date were 7.7 and 7.3 in the AV-OVA-1 arm, and 7.9 and 7.7 in the MC arm. Mean and median durations from randomization to the first treatment date were 1.6 and 1.4 months in the AV-OVA-1 arm (range 1.0 to 3.4) and 1.6 and 1.2 months in the MC arm (range 1.0 to 5.2).

Inclusive treatment dates with study agents were August 2018 through February 2022. Per direction from its Board of Directors, the sponsor terminated the study prematurely because of the ongoing SARS-CoV-2 pandemic and associated declining rate of enrollment, a concern that survival may have improved for all patients because of increased use of PARP inhibitors, and competing financial priorities. After enrollment was closed, patients who were undergoing treatment were allowed to complete all vaccine injections. Those who had already undergone successful leukapheresis and had a cell line available were then randomized and allowed to complete therapy with study agents. Long-term follow-up was discontinued after study closure; so, subsequent patients were only followed for 28 days after the last dose. Thus, for patients who received all eight injections, the minimum follow-up from the randomization date was about nine months, and the longest follow-up was 30 months.

### 3.2. Patient Characteristics and Other Therapies

Table 1 shows patient characteristics and cancer treatments given before and/or concurrently with the study agents. There were no significant differences in the proportions of patients with various characteristics, including stage at diagnosis (*p* = 0.169), and proportion greater than 60 years of age (*p* = 0.354). A total of 75% had a high-grade serous histology; 71% had stage 3 disease. Two-thirds were treated with both neoadjuvant and adjuvant platinum-based chemotherapy. After debulking surgery, 40% were totally debulked, 33% were optimally debulked, 7% were suboptimally debulked, and for 20% post-surgery information was not provided. A total of 20% had persistent disease at the time of stratification for randomization.

Most patients received neoadjuvant chemotherapy. A total of 28 (62.2%) received three cycles of chemotherapy pre-debulking plus three or more post-interval debulking; one patient received six cycles of chemotherapy and then underwent definitive surgery. A total of 16 patients (35.6%) received adjuvant platinum-plus-taxane chemotherapy after primary debulking surgery. A total of 44.2% received PARP inhibitors during primary chemotherapy; 18.2% received a PARP inhibitor concurrent with the study agent. Of the 23 who received a PARP inhibitor, 14 received a PARP inhibitor only during primary chemotherapy, five received it with both primary chemo and vaccine, and four did not receive PARP concurrently with a study agent. Pre-randomization, there was little difference in anti-cancer agents received, but during the study, more than twice as many patients in the AV-OVA-1 arm received systemic anti-cancer treatment (71.4% vs. 31.2%, *p* = 0.013). Presumably, the reason for giving concurrent therapy was that cancer had progressed or persisted despite standard therapy, which suggests that patients in the AV-OVA-1 arm may have had a worse prognosis at the time of randomization.

### 3.3. Study Product Manufacturing Success

Despite the complex logistics, success rates were high for the manufacturing of intermediate and final cell products. As shown in the CONSORT Diagram (Figure 2), there was a 97% success rate for establishing tumor cell lines. Table 2 shows the number of cells manufactured for the intermediate and final study products. The goal of 100 million cells was achieved for all but three tumor samples. The microsphere technique using proprietary stem cell media was used to enrich for stem cells, but short-term expansion often required the addition of fetal bovine serum. The average number of days in culture was 25.0 for tumors from patients eventually randomized to AV-OVA-1 and 26.6 days for those randomized to MC. Tumors were in culture for 28 days or less in 23/29 (79%) in the AV-OVA-1 arm and 13/16 (81%) in the MC arm. Cultured cells had high expression of the stem cell markers nestin and CD44. The most striking difference supporting enrichment for early progenitor cells versus more differentiated cells was the phenotypic data for MUC-1 and CA-125 expression. Phenotypic data for MUC-1 and CA-125 expression were available for 33 cell lines. MUC-1 expression was present on 99 to 100% of cells for 26/33 (79%), 90% or more for 29/33 (88%), and 80% or more cells for 31/33 (94%); 69% and 60% of cells were MUC-1 positive in the other two cell cultures. In contrast, even though based on pathology reports, 90% of the ovarian cancers expressed high levels of the differentiation marker CA-125, it was expressed on only 0 to 1% of cells for 32/33 cell lines (97%), and the expression was on only 3% of cells in the positive sample.

The success rate for collecting an adequate number of MCs per patient was 94%, but three patients needed a second pheresis procedure to achieve adequate numbers. Greater than 450 million viable monocytes were cryopreserved for potential differentiation into DC for 44/45 patients (98%). Greater than one million cells/dose were cryopreserved for 29/29 (100%) AV-OVA-1 products, with more than one million viable cells post-cryopreservation for 27/29 (93%) AV-OVA-1 products.

Of the 47 patients who had both a cell line and sufficient monocytes, one withdrew, and one was ineligible based on her cancer stage. The remaining 45 patients were randomized, 29 to AV-OVA-1 and 16 to MC. However, during analysis after concluding the trial, it was discovered that an autologous AV-OVA-1 product was manufactured erroneously, and all eight AV-OVA-1 doses were injected into a patient who was randomized to MC. Thus, in terms of actual treatment products, 15 patients received MC, 29 received AV-OVA-1, and one AV-OVA-1 product was not used due to contamination.

### 3.4. Safety

The 45 randomized patients received 333 injections. There were no differences in the average number of injections: 7.4 in the AV-OVA-1 arm and 7.3 in the MC arm, or in the proportions who received all eight possible doses: 26/28 (92.9%) in the AV-OVA-1 arm and 14/16 (88%) in the MC arm. All eight injections were administered to 27/29 (93.1%) patients treated with AV-OVA-1 and 13/15 treated with MC (86.7%).

Table 3 summarizes AE frequency by type and study agent received. Treatments were well-tolerated; no patients discontinued study therapy because of toxicity. The vast majority of AE were mild to moderate in severity, brief in duration, and self-limited in that specific medical measures were not required for resolution. A total of 28/29 AV-OVA-1-treated patients and 15/15 MC-treated patients reported at least one AE. There was a total of 449 TEAEs recorded for the 44 patients who received at least one dose of a study product: 308 in the 29 AV-OVA-1-treated patients and 141 AE in the 15 MC-treated patients. The frequency and severity of AE were similar regardless of the study agent. The number of AE per patient ranged from 0 to 26 (mean 10.6, median 11) with AV-OVA-1 treatment and from 3 to 16 (mean 9.6, median 8) with MC treatment (*p* = 0.56). AEs were mainly attributed to underlying ovarian cancer and other therapies. The most common TEAE were local injection site reactions (ISRs) (redness, swelling, discomfort), general systemic symptoms (fatigue, flu-like symptoms, chills, fever, and body aches), and musculoskeletal (bone pain, joint pain, muscle aches). There were no differences between the study arms in the frequency or severity of AEs. Both study products were admixed with GM-CSF shortly before each injection and s.c. GM-CSF injections are known to produce ISR in a high proportion of patients. There was only one serious adverse event (SAE) attributed to a study agent; it occurred in a patient in the MC control arm who was hospitalized for high-dose corticosteroids to treat refractory urticaria that occurred after the eighth injection.

Table 4 summarizes the highest grade AE experienced in each study arm. There were no differences in toxicity grades between the two study products; the median AE grade was 1 in both arms. The average grade AE for the 308 AE that occurred in AV-OVA-1-treated patients was 1.31 compared to 1.29 for the 141 AE that occurred in MC-treated patients (*p* = 0.68). There was no difference in the distribution of AE by severity grade or highest grade AE per patient, nor by whether study treatment was given concurrently with other treatments, typically chemotherapy, bevacizumab, PARP inhibitor, and/or anti-PD-1. There was an apparent difference in the frequency of hematologic events between the two treatment products (30/308 vs. 5/141), but this is misleading because two AV-OVA-1-treated patients accounted for 18 of the 30 hematologic AE (9 each), all of which were attributed to concurrent chemotherapy. The average numbers of AEs were 1.5 for AV-OVA 1 plus GM-CSF, 2.3 for other therapy plus AV-OVA plus GM-CSF, 2.0 for MC plus GM-CSF, 2.0 for other therapy plus MC plus GM-CSF, 1.8 for either study treatment, and 2.2 for either study treatment plus other therapy. Most patients did not receive specific treatment for AE. For example, 27/44 patients experienced 78 ISRs; no treatment was administered for 64/78 (82%). Drugs administered for AE included acetaminophen (n = 5), corticosteroids (n = 5), anti-histamines (n = 4), and non-steroidal anti-inflammatory agents (n = 3). Corticosteroids were administered to three AV-OVA-1-treated patients: one grade 3 “unrelated” arthritis after dose 5 attributed to concurrent pembrolizumab, one grade 1 “unrelated” arthritis after dose 6, and one “related” grade 2 Sjogren’s syndrome after dose 4. Corticosteroids were administered to two MC-treated patients: one “possibly related” grade 3 urticaria and rash after dose 8, and one “unrelated” grade 3 bilateral uveitis after dose 3 that was attributed to concurrent pembrolizumab.

Surprisingly, there was a substantial difference in AE distribution among the three AE study agent attributions. Inexplicably, investigators classified a higher proportion of AE “related or possibly related” (141/308 vs. 96/141, *p* < 0.0001), or “related” (87/308 vs. 57/141, *p* = 0.010) in patients treated with MC plus GM-CSF compared to those treated with AV-OVA-1 plus GM-CSF. There were no grade-4 TEAEs considered “related” or “possibly related” to a study agent. Among the 29 AV-OVA-1-treated patients, there were 16 grade-3 AEs, 14 considered “unrelated,” 1 thrombocytopenia considered “related” to treatment, and 1 hypertension considered “possibly related.” Among the fifteen MC-treated patients, there were eight grade 3 AEs, with five considered “unrelated” to study treatment, one thrombocytopenia considered “related,” and one patient reported headache and rash that were categorized as “possibly related”.

### 3.5. Efficacy

#### 3.5.1. Overall Survival

**Figure 3:**
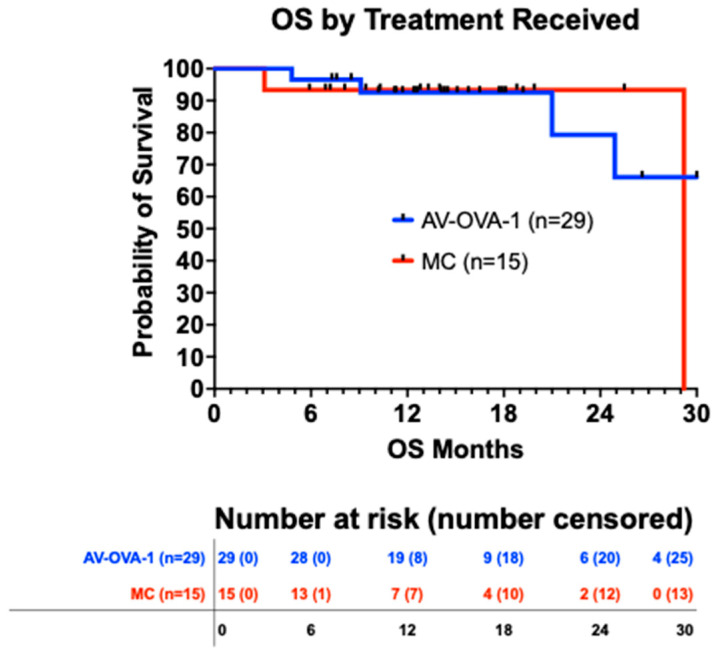
Overall survival from date of randomization by treatment received (*p* = 0.734). The median OS was undefined for AV-OVA-1 (four deaths) vs. 29.2 months for MC (two deaths). The 12-month and 24-month OS rates were 92.5% vs. 93.3% and 79.3% vs. 93.3%, respectively.

For all 45 patients, the median OS was 30.0 months from randomization (95% CI 25.0 to 31.1 months) and 36.5 months from initial surgery (95% CI 28.8 to 39.7). There was no difference in OS by the intent-to-treat analysis nor by the study treatment actually received (Figure 3). The survival curves for both analyses were virtually identical. However, 43 deaths were needed for the planned OS evaluation, and after a maximum of 2.5 years of follow-up, there were only six deaths, four in the AV-OVA-1 arm and two in the MC arm.

#### 3.5.2. Progression-Free Survival

**Figure 4:**
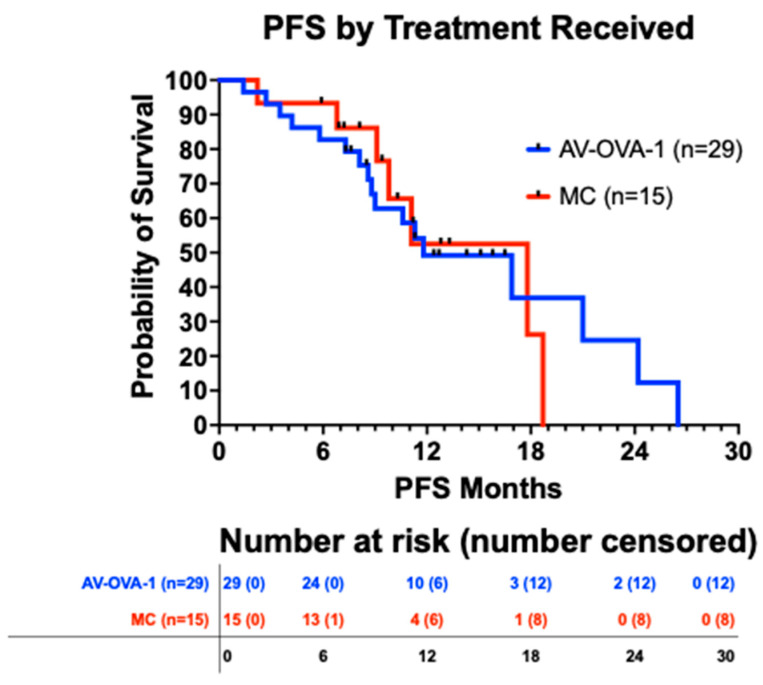
Progression-free survival from date of randomization by treatment received (*p* = 0.919). The medians were 11.8 months (17 events) vs. 17.8 months (7 events). The 12-month and 24-month PFS rates were 49.2% vs. 52.5% and 24.6% vs. 0%, respectively.

For all 45 patients, based on 24 events, the median PFS was 16.9 months (95% CI 10.0 to 18.7) from randomization and 18.8 months (95% CI 16.4 to 25.4) from initial surgery. There was no difference in the PFS curves by the intent-to-treat analysis nor by the study treatment actually received (Figure 4). There was no difference in PFS based on whether PARP inhibitors were administered. The 23 patients who received a PARP inhibitor had a median PFS of 18.7 months (95% CI 9.6 to 18.7 months) compared to 11.3 months (95% CI 8.5 to 17.7 months) for 21 patients who did not receive a PARP inhibitor (*p* = 0.557).

#### 3.5.3. Immune Response

**Figure 5:**
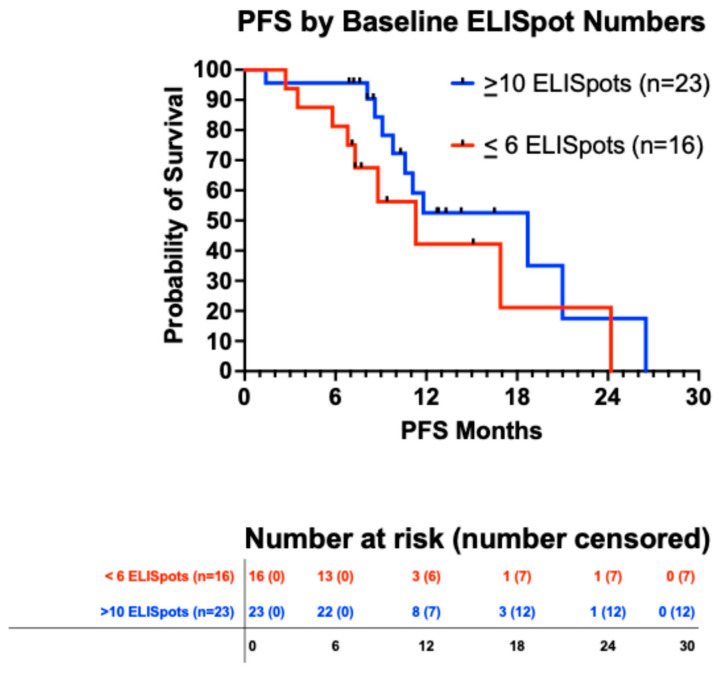
Progression-free survival based on ELISpot numbers at baseline: high (≥10, median = 61, mean = 90.1 ± 14.4 SEM, range 10 to 298) vs. low (≤6, median = 2, mean = 2.3 ± 0.5 SEM, range 0 to 6). The PFS medians were 18.7 months (11 events) vs. 11.3 months (9 events), *p* = 0.175.

At baseline, for all patients for whom data were available (n = 39), the median ELISpot number was 22, the mean 54.1 ± 11.3 SEM, and the range was 0 to 298. Figure 5 shows that the 23 patients with a baseline ELISpot count of 10 or greater may have had a slightly better PFS than the 16 patients who had an ELISpot count of six or less (*p* = 0.175).

**Figure 6:**
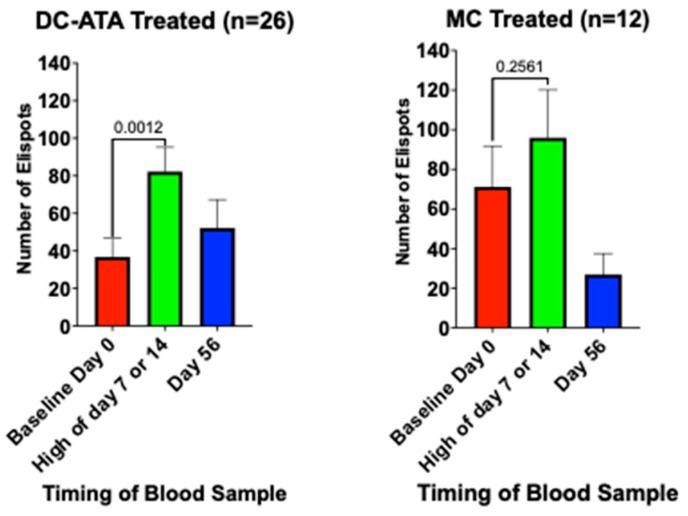
T cell immune response by treatment arm, before and after the first two vaccinations, based on the number of gamma interferon ELISpots.

There were 38 patients for whom ELISpot numbers were available both at baseline before the first injection and just before the injections one and two weeks later. At baseline, IFN-γ-ELISpots were 10 or greater in 13/26 (50%) AV-OVA-1-treated patients compared to 10/13 (77%) MC-treated patients (*p* = 0.18). At baseline, the mean number of ELISpots in the 26 AV-OVA-1-treated patients was 36.8 ± 9.9 SEM (median = 13, range 0 to 163) compared to a mean of 88.6 ± 26.8 (median = 61, range 0 to 298) in the 12 MC-treated patients (*p* = 0.029). Figure 6 shows the changes in ELISpots before and after the first two vaccinations (days 7 and 14). Shortly after starting vaccination, based on day-7 or day-14 counts, ELISpots were positive for 22/24 (92%) in the AV-OVA-1 arm and 11/13 (85%) in the MC arm. ELISpot numbers in the AV-OVA-1 arm more than doubled, but there was no change in the MC control arm. The six patients in the AV-OVA-1-treated group who did not have at least seven ELISPOTS at either day 7 or day 14 had PFS of 5.8, 7.3, 11.3, and 21.0 months, while the two patients in the MC-treated group who did not have at least seven ELISPOTS at either day 7 or day 14 had PFS of 6.8 and 18.7 months. There was insufficient residual irradiated TIC lysate for in vitro ELISPOT testing.

## 4. Discussion

This randomized, multicenter phase 2 trial confirmed the feasibility of reliably producing DC-ATA specifically for ovarian cancer, established the absence of significant toxicity associated with serial s.c. injections of AV-OVA-1, and demonstrated induction of T-cell responses that were not seen in the control arm. Premature closure of the trial limited the ability to draw definitive conclusions regarding efficacy based on PFS and OS.

There are significant logistical challenges in the manufacturing of personal DC-ATA vaccines. Personal study product manufacturing and administration required: (1) coordinating tumor collection during laparotomy and shipping to the manufacturing site, (2) a successful short-term culture of self-renewing tumor cells, (3) collection of sufficient PBMC numbers by leukapheresis and shipping of PBMC to the manufacturing site, (4) successful differentiation of monocytes into DC, (5) manufacturing of the final product by incubating DC with the lysate of autologous irradiated self-renewing TICs, (6) successful quality control testing, and [7] coordinated shipping of individual doses to treatment locations and subsequent vaccine injections within five hours after the study product was thawed. Despite these obstacles, feasibility and reproducibility were confirmed in a multicenter trial that required shipping of products to and from various geographical locations. AV-OVA-1 was reliably manufactured and delivered for injection, with high success rates for growing short-term tumor cell lines, generating DC from PBMC, and generating final AV-OVA-1 treatment products. The success rates were virtually identical to those reported in a single-arm trial of patient-specific DC-ATA in patients with primary GBM [33].

As was observed in our other DC-ATA trials [29,30,31,32,33], AV-OVA-1 was well-tolerated with no patients stopping treatment because of AEs, no SAEs attributed to the vaccine, local ISRs the most common AEs, the vast majority of AEs being mild to moderate in severity, brief, and self-limited, and more than 90% of planned vaccine doses injected. The frequency and severity of AEs were similar between the two study arms, which suggests treatment-related AEs are likely due to adjuvant GM-CSF. AV-OVA-1 injections were associated with enhanced immune responses compared to baseline numbers of ELISpots. This was not seen in the MC control arm. Increased immune response following DC-ATA injections was also demonstrated in a melanoma randomized trial [32,40]. Increases in cellular immune response have been demonstrated following other vaccines [41], including other patient-specific DC vaccines [23,24,27]. However, such immune changes have not been predictive of clinical outcomes.

The available data did not suggest a difference in OS between AV-OVA-1 and MC, but because so few deaths had occurred due to the premature trial closure, this was inconclusive. Given the study design and assumptions, six deaths had only 30% power to detect an HR = 0.50. With only six deaths, the desired 80% power would have necessitated an HR = 0.15, an 85% reduction in the risk of death. It is hard to put the survival from initial surgical diagnosis (36.8 months OS and 18.8 months PFS) into perspective compared to other trials because 25 patients for a whom a cell line was produced were not randomized, and before randomization about 20% of patients in the current trial had disease that was not controlled with primary platinum-taxane chemotherapy, more than 40% received a PARP inhibitor concurrently with primary chemotherapy, and more than 40% received bevacizumab.

The major strengths of this study are (1) enrollment of patients from multiple clinical sites, (2) eligibility criteria that assured that a better prognostic population was not created unintentionally, (3) enrollment of patients representative of ovarian cancer patients, (4) the use of blinding and randomization with a contemporary control group, (5) OS as the primary endpoint, (6) the high proportion of patients who were able to be randomized about seven months after their initial surgery, (7) the most frequent reasons for not proceeding with protocol prescribed procedures were patient withdrawal or inappropriate histology or stage rather than issues related to vaccine production. Premature closure is the major weakness of the study. This meant that the study arms were relatively small and left the study underpowered to test its primary objective. Another weakness was the inability to perform ELISpot tests that tested the addition of irradiated tumor cell lysate in vitro.

At this time, there are no vaccine therapies approved for patients with advanced ovarian cancer. As reviewed elsewhere, others have and are also investigating DC vaccines for ovarian cancer [42,43,44,45]. Earlier strategies focused on autologous DCs loaded with antigens shared among patients, such as MUC1 [39], p53 peptide [46], Her2/neu, hTERT, and PADRE peptides [47], WT-1 [48], the antigen folate receptor alpha [49], and the use of autologous DCs loaded with antigens from allogeneic ovarian cancer cell lines [50]. However, efforts are increasingly being focused on ATA [51,52,53].

## 5. Conclusions

This study established that the manufacturing of AV-OVA-1 for ovarian cancer patients can be reliably performed and that treatment with the personal AV-OVA-1 vaccine is well tolerated by ovarian cancer patients. Treatment with AV-OVA-1 is associated with increased T-cell responses, but additional studies are needed to address the impact on PFS and OS.

## Figures and Tables

**Table 1:** Patient characteristics and other therapies by study arm assignment.

Variable	AV-OVA-1 (n = 29)	MC (n = 16)
Age		
Median	61 years	60 years
Range	42 to 78	39 to 84
<50	3 (10.3%)	3 (18.8%)
50–59	9 (31.0%)	5 (31.2%)
60–69	16 (55.2%)	4 (25.0%)
70–79	3 (10.3%)	2 (12.5%)
>80	0	2 (12.5%)
Race/Ethnicity		
Asian	2 (6.9%)	1 (6.2%)
Black	0 (0%)	0 (0%)
Hispanic	4 (13.8%)	3 (18.8%)
White	23 (79.3%)	12 (75%)
ECOG PFS at baseline		
0	19 (65.5%)	11 (68.8%)
1	9 (31.0%)	4 (25.0%)
2	1 (3.4%)	1 (6.2%)
Stage at Diagnosis		
Stage 3	23 (79.3%)	9 (56.2%)
Stage 4	6 (20.7%)	7 (43.8%)
Tissue of Origin		
Ovary	21 (72.4%)	13 (68.8%)
Fallopian Tube	6 (20.7%)	2 (12.5%)
Primary Intraperitoneal	2 (6.9%)	1 (6.2%)
Histology		
High-Grade Serous	22 (75.9%)	12 (75.0%)
Papillary Serous	1 (3.4%)	1 (6.2%)
Serous/Endometrioid	2 6.9%)	0 (0%)
Adenocarcinoma	1 (3.4%)	0 (0%0
Poorly Differentiated	3 (10.3%)	1 (6.2%)
Clear Cell	0 (0%)	2 (12.5%)
Extent of surgical debulking		
No residual disease	11 (37.9%)	7 (43.8%)
Optimally debulked ^1^	10 (34.5%)	5 (31.2%)
Suboptimally debulked ^2^	1 (3.4%)	2 (12.5%)
Not available	6 (20.7%)	2 (12.5%)
At randomization		
No Evidence of Disease	23 (79.3%)	13 (81.2%)
Residual Disease	6 (20.7%)	3 (18.8%)
Time from Surgery to Randomization		
Mean months	6.1	6.4
Median months	5.9	6.3
Range in months	3.2 to 15.1	3.1 to 13.4
Time from Surgery to First Injection		
Mean months	7.1	7.9
Median months	7.3	7.7
Range in months	4.6 to 16.3	4.5 to 14.6
Time from Randomization to First injection		
Mean months	1.6	1.6
Median months	1.4	1.2
Range in months	1.0 to 3.4	1.0 to 5.2
Primary Platinum-based Chemotherapy	AV-OVA-1 (n = 29)	MC (n = 16)
Neoadjuvant + adjuvant	20 (69.0%)	10 (62%)
Adjuvant	9 (31.0%)	6 (38%)
Treatment Agents		
Taxane	28 (96.4%)	16 (100%)
Platinum	29 (100%)	16 (100%)
PARP Inhibitor	12 (41.4%)	7 (43.8%)
Bevacizumab	11(37.9%)	7 (43.8%)
Anti-PD-1	1 (3.4%)	1 (6.2%)
Doxil	0	1 (6.2%)
Gemcitabine	0	1 (6.2%)
Therapy concurrent with vaccine therapy	AV-OVA-1 (n = 28)	MC (n = 16)
Any concurrent therapy	20 (71.4%)	5 (31.2%)
Taxane	1 (3.6%)	0 (0%)
Platinum	3 (10.7%)	0 (0%)
Doxil	4 (14.3%)	0 (0%)
Bevacizumab	8 (28.6%)	2 (12.5%)
Campothecin	1 (3.6%)	0 (0%)
Gemcitabine	2 (7.2%)	1 (6.2%)
PARP Inhibitor	7 (25.0%)	2 (12.5%)
Anti-PD-1	1 (3.6%)	1 (6.2%)
Anti-Estrogen	1 (3.6%)	0 (0%)

PFS = performance status; PARP = poly (ADP-ribose) polymerase; PD-1 = programmed death molecule-1. ^1^ Optimally debulked = residual disease ≤ 1 cm in greatest diameter. ^2^ Sub-optimally debulked = residual disease > 1 cm in greatest diameter.

**Table 2:** Characteristics of intermediary and final products manufactured.

Variable	Randomized to AV-OVA-1 (n = 29)	Randomized to MC (n = 16)	*p* Value
Tumor cell line			
Mean days in culture	24.1	24.8	0.87
Median days in culture	20.0	26.5	
Range of days in culture	8 to 62	10 to 67	
In culture 28 days or less	23 (79.3%)	13 (81.2%)	
Tumor cells lysed			
Mean × 10^6^	66.7	45.0	0.071
Median × 10^6^	81.7	41.0	
Range × 10^6^	1.8 to 135	1.1 to 96.8	
10 million or more	26 (89.7%)	11 (68.8%)	
Monocytes cryopreserved			
Mean × 10^9^	1.70	1.13	0.022
Median × 10^9^	1.56	1.01	
Range × 10^9^	0.63 to 3.80	0.25 to 2.11	
450 million or more	29 (100%)	15/16 (93.8%)	
Final AV-OVA-1 product	AV-OVA-1 (n = 29)	MC (n = 15)	
Mean cell number × 10^6^	95.7	N/A	
Median cell number × 10^6^	66.8	N/A	
Cell number range × 10^6^	17.8 to 508	N/A	
10 million or more cells	29 (100%)	N/A	
Viable cells cryopreserved	DC-ATA (n = 29)	MC (n = 15)	
Mean × 10^6^ per dose	8.2	12.0	
Median × 10^6^ per dose	6.0	9.2	
Range × 10^6^ per dose	2.0 to 27.0	8.0 to 24.6	
1 million or more per dose	29 (100%)	15 (100%)	

AV-OVA-1 = autologous dendritic cell (DC) autologous tumor antigen (ATA) vaccine; MC = autologous monocytes; DC = dendritic cells; ATA = autologous tumor antigens; N/A = not applicable.

**Table 3:** Most frequent adverse events (AEs): percentages of patients experiencing a specific AE, and specific AEs as a percentage of AEs displayed by treatment received, and by body system and event, regardless of grade.

	AV-OVA-1	MC	AV-OVA-1	MC	AV-OVA-1	MC
Body System	% of 29 Pts	% of 15 Pts	% of All 308 AE	% of All 141 AE	% of Related 87 AE	% of Related 57 AE
INJECTION SITE REACTION	58.6	66.7	16.2	19.9	50.6	42.1
GENERAL	69.0	60.0	18.2	17.7	29.6	20.5
Fatigue	44.8	33.3	5.8	5.0	9.3	12.8
Fever	17.2	13.3	3.2	3.5	5.6	0.0
Body aches	20.7	6.7	1.0	0.7	3.7	2.6
Chills	10.3	20.0	2.6	3.5	1.9	2.0
Flu-like symptoms	13.8	6.7	3.5	4.3	3.7	0
Malaise (lethargy)	10.3	6.7	1.0	0.7	3.7	2.6
DERMATOLOGIC	41.4	46.7	8.4	9.9	12.6	12.3
Pruritis	27.6	20.0	4.5	3.5	5.3	8.3
Rash	13.8	26.7	1.6	5.0	5.3	2.1
Hives (urticaria)	6.9	13.3	1.0	1.4	0.0	1.8
MUSCULOSKELETAL	69.0	60.0	13.3	19.9	11.5	12.3
Muscle aches, myalgias	20.7	53.3	2.6	7.8	4.6	8.8
Joint pain, discomfort, arthralgias	24.1	13.3	3.2	2.1	3.4	0
Back pain	10.3	20.0	1.3	3.5	0.0	0.0
Bone pain	17.2	6.7	1.6	1.4	2.3	3.5
GASTROINTESTINAL	65.5	46.7	10.4	12.8	0.0	1.8
Abdominal or pelvic pain	34.5	33.3	3.2	4.9	0.0	0.0
Constipation	17.2	20.0	1.6	3.5	0.0	0.0
Nausea	24.1	20.0	2.3	2.1	0.0	0.0
NEUROLOGIC	41.4	26.7	6.2	5.7	1.1	0.0
Headache	37.9	20.0	4.2	5.0	1.1	0.0
HEMATOLOGIC	20.7	13.3	9.7	3.5	1.1	1.8
Thrombocytopenia/petechiae	13.8	6.7	2.6	1.4	1.1	1.8
Neutropenia	13.8	0.0	4.2	0.0	0.0	0.0
Anemia	6.9	6.7	1.6	2.1	0.0	0.0
EAR NOSE AND THROAT	41.4	20.0	6.5	2.1	1.1	0.0
Nasal congestion, rhinitis, post-nasal drip	17.2	0.0	2.6	0.0	0.0	0.0
Oral pain (tongue or mouth, ulceration)	10.3	0.0	1.0	0.0	0.0	0.0
CARDIOVASCULAR	24.1	0.0	2.9	0.0	1.9	0.0
Hypertension	13.8	0.0	2.0	0.0	1.9	0.0
PSYCHOLOGIC	17.2	13.3	2.3	2.1	0.0	0.0
Depression, mood dysphoria	3.4	13.3	0.3	1.4	0.0	0.0
Insomnia	6.9	6.7	0.6	0.7	0.0	0.0
PULMONARY	20.7	0.0	1.9	0.0	0.0	0.0
Cough	13.8	0.0	1.3	0.0	0.0	0.0
OCULAR	13.8	6.7	1.3	2.1	1.1	0.0
Blurred vision	10.3	6.7	0.6	1.4	1.1	0.0

**Table 4:** Highest grade adverse event experienced by each patient, and numbers and percentages of all adverse events by grade and study agent received.

	AV-OVA-1 (n = 308)			MC (n = 141)			Total (n = 449)		
Grade	Number and Percent of Highest AE per Patient	Number and Percent of All AE	Mean AE per Patient	Number and Percent of Highest AE per Patient	Number and Percent of All AE	Mean AE per Patient	Number and Percent of Highest AE per Patient	Number and Percent of All AE	Mean AE per Patient
1	5 (17%)	229 (74%)	7.9	5 (33%)	108 (77%)	7.2	10 (23%)	337 (75%)	7.7
2	13 (45%)	62 (20%)	2.1	5 (33%)	25 (18%)	1.7	18 (41%)	87 (19%)	2.0
3	9 (31%)	16 (5%)	0.6	5 (33%)	8 (6%)	0.5	14 (32%)	24 (5%)	0.5
4	1 (3.4%)	1 (0.3%)	0.03	0	0	0	1 (2.3%)	1 (0.2%)	0.02
Total	28 (97%)	308 (100%)	10.6	15 (100%)	141 (100%)	9.4	43 (98%)	449 (100%)	10.2

## Data Availability

Data are available upon reasonable written request for legitimate scientific research. Requests should be directed to ROD.
